# Contrasting vegetation height and elevation shape near-surface thermal responses more strongly than slope orientation during summer in northern Patagonia

**DOI:** 10.1007/s00484-026-03254-w

**Published:** 2026-06-29

**Authors:** Jonas Fierke, Juan Haridas Gowda, Gastón Mauro Díaz, Philipp Koal, Helge Walentowski, Martin Kappas, Birgitta Putzenlechner

**Affiliations:** 1 Institute of Geography, University of Göttingen, Goldschmidtstraße 3, 37077 Göttingen, Germany; 2 INIBIOMA, Universidad Nacional del Comahue-CONICET, Quintral 125, Bariloche, 8400 Argentina; 3 Patagonian Andean Forest Research and Extension Center (CIEFAP), Department of Geomatics, Ruta 259 16.24, Esquel, 9200 Argentina; 4 Forestry Research and Competence Centre, ThüringenForst AöR, Jägerstraße 1, 99867 Gotha, Germany; 5 Faculty of Resource Management, University of Applied Sciences and Arts, Daimlerstraße 2, 37075 Göttingen, Germany; 6 Competence Center Landscape Resilience, University of Göttingen, Büsgenweg 1, 37077 Göttingen, Germany

**Keywords:** Microclimate, Temperate mountain forests, Near-surface temperature, Linear mixed effects models, Patagonia

## Abstract

Forests in temperate mountain regions are increasingly exposed to extreme heat. Near-surface microclimates, shaped by vegetation and topography, influence forest regeneration, fuel dynamics, and disturbance feedbacks. We examined how daily warming rates, maximum temperatures, and cooling rates differ between microsites contrasting in vegetation height, elevation, and slope orientation during summer, and whether these differences increase under higher summer temperatures. We deployed a network of temperature loggers in the Río Manso Valley (northern Patagonia), recording summer temperatures at 15-minute intervals. Using a paired sampling design, we compared six environmental contrasts representing forest versus shrubland, low versus high elevation, and north- versus south-facing slopes. Following a contrast-wise approach, we used linear mixed-effects models to quantify daily differences in thermal metrics between paired microsites and assessed how these differences scaled with maximum daily temperature at the warmer site within each pair. Summer thermal sensitivity varied strongly across environmental contrasts. Differences associated with vegetation height and elevation were larger and more consistent than those associated with slope orientation. Across all contrasts, differences in warming rates, maximum temperatures, and cooling rates increased with rising summer temperatures, indicating that microclimatic divergence among microsites amplifies under heat extremes. By quantifying near-surface thermal sensitivity across paired environmental contrasts, our study provides empirical information relevant for modelling microclimate-sensitive ecological processes under climate change, particularly in comparable temperate mountain systems.

**Supplementary information:**

The online version contains supplementary material available at 10.1007/s00484-026-03254-w.

## Introduction

Forests in temperate mountain regions are increasingly exposed to climate-driven changes in disturbance regimes, including more frequent and severe droughts and wildfires (Bebi et al. 2017; Kulakowski et al. 2017; Albrich et al. 2020). These dynamics are altering forest structure, composition, and post-disturbance recovery processes, with important implications for ecosystem functioning and resilience (Sommerfeld et al. 2018). While these processes operate at broad spatial scales, their local expression depends strongly on fine-scale environmental conditions, with near-surface microclimates shaped by vegetation and topography (Carnicer et al. 2021).

Vegetation structure and topography modulate near-surface temperature by altering incoming and outgoing radiation, canopy shading, evapotranspiration, and air flow, thereby shaping the local surface energy balance and creating heterogeneous microclimatic conditions (Geiger et al. 2009; De Frenne et al. 2021). In temperate mountain landscapes experiencing increasing heat and drought stress, near-surface temperature dynamics are increasingly recognised as an important component of the environmental context influencing forest recovery and disturbance-related processes. Microclimatic conditions during and after disturbance shape regeneration outcomes by influencing temperature extremes experienced at the seedling to sapling level. Certain microsites, such as those beneath forest canopies, can reduce thermal stress during sensitive life stages like germination and early establishment (Marsh et al. 2022a, b; Hill et al. 2024). In contrast, unsheltered locations may favour light-demanding species by offering greater irradiance and warmer conditions, but only under adequate moisture availability (Espinosa del Alba et al. 2025). Beyond regeneration, near-surface temperatures also affect surface fuel conditions: 2008cooler and moister microsites reduce fuel drying, potentially lowering ignition risk and slowing fire spread during hot and dry periods (Barberá et al. 2023). These roles are further reinforced by vegetation–microclimate feedbacks. Even modest increases in vegetation height or density can dampen thermal variability, creating a self-reinforcing cooling effect (D’Odorico et al. 2010, 2013). Understanding how vegetation and topography interact to shape near-surface temperatures is therefore essential for anticipating landscape trajectories in a warming, fire-prone climate.

Recent studies have advanced our understanding of forest microclimates by collecting fine-scale temperature data along gradients of vegetation structure, elevation, or topography (Atkins et al. 2023; Máliš et al. 2023; Jia et al. 2024). While these efforts have yielded important insights, direct comparisons across multiple environmental contrasts within the same basin remain relatively scarce. Moreover, thermal sensitivity is frequently characterized using temperature extremes, such as daily maxima, whereas temporal features like the rate and timing of warming and cooling have received comparatively less attention, despite their ecological relevance (Hill et al. 2024). Previous findings also suggest that microclimatic responses to heat are temperature-dependent, with differences among sites becoming more pronounced under extreme conditions (Davis et al. 2019; De Frenne et al. 2019; Starck et al. 2025). This underlines the need for comprehensive assessments that span multiple environmental contrasts and capture both thermal amplitude and diurnal dynamics. Such efforts are particularly important in mountainous landscapes, where complex terrain creates high microclimatic variability and may reveal context-dependent thermal responses.

To address these gaps, we examine how near-surface thermal sensitivity differs across multiple paired environmental contrasts in a temperate mountain landscape of northern Patagonia, Argentina. With paired sites that contrast in elevation, slope orientation, and vegetation height, including forest stands and structurally open sites, our methodology quantifies differences in warming rates, daily maximum temperatures, and cooling rates during summer. This allows us to assess how near-surface thermal conditions respond to rising temperatures across forest and shrubland of high conservation value, undisturbed by economic activities.

Northern Patagonia offers a particularly relevant context for this analysis because, while much of the region remains dominated by continuous native forests, recent and projected increases in heat and drought raise concerns about growing wildfire vulnerability (Kitzberger et al. 2022). Understanding how local microclimatic conditions are structured by vegetation and topography is of importance to predict how forest regeneration and disturbance feedbacks will unfold under future climate extremes. Knowledge gains in well-conserved ecosystems can shed light on what are the better management practices to achieve resilient landscapes.

In this study, we examine how near-surface thermal dynamics vary across paired environmental contrasts in a temperate mountain landscape of northern Patagonia. Specifically, we address the following questions: (1) How do daily warming rates, maximum temperatures, and cooling rates differ across paired environmental contrasts in elevation, slope orientation, and vegetation height during summer? (2) How does the magnitude of these differences change with increasing summer temperatures? By comparing topographic and vegetation-related contrasts within a single mountain landscape, our study clarifies how near-surface thermal dynamics vary across space and temperature, providing empirical constraints for microclimate modelling under climate change in complex mountain landscapes.

## Materials and methods

### Study area

The study was conducted in the Río Manso Valley, northern Patagonia, Argentina (41.60° S, 71.56° W), approximately 5 km west of the town of Río Villegas. This temperate mountain landscape is characterized by steep elevation variation, varying slope orientations, and a mosaic of vegetation types shaped by both natural disturbance dynamics and historical land use (Gowda et al. 2012; Kitzberger 2012). Over short distances, the valley transitions between dense *Nothofagus pumilio* forests, structurally open shrublands dominated by *Nothofagus antarctica*, and mixed formations, creating high microclimatic and structural heterogeneity. The region experiences a marked seasonality in temperature and precipitation, with increasingly frequent summer heat extremes and wildfire activity (Holz et al. 2012; Kitzberger et al. 2022), making it a representative setting for studying interactions between topography, vegetation structure, and climate stressors.

### Microclimatic sampling

To assess near-surface thermal variation across paired environmental contrasts, we deployed a network of 22 TOMST TMS-4 Standard loggers (Wild et al. 2019), each installed along a valley transect of Río Manso Inferior Valley. All sensors recorded temperatures in 15-minute intervals from February 15, 2024, to February 14, 2025, and were shielded from direct solar radiation to ensure consistent and comparable microclimatic measurements. The loggers record temperature at three levels (–6 cm below surface, + 2 cm above the surface, and + 15 cm above surface), as defined by the logger design. We focused our analysis on the 15 cm measurements, as they provide a consistent representation of near-surface temperature across structurally heterogeneous sites. This choice reflects both the practical difficulty of maintaining a consistent sensor height at 2 cm across structurally complex vegetation (Aalto et al. 2022), where vegetation cover and litter can vary strongly at small spatial scales, and the limited sensitivity of subsurface temperatures (–6 cm) to short-term warming and cooling dynamics near the surface. The 15 cm level therefore represents a practical compromise between capturing near-surface thermal dynamics relevant for ecological processes and ensuring comparability across sites.

The study was designed to capture three key environmental contrasts: (i) elevation (low vs. high), (ii) slope orientation (north- vs. south-facing), and (iii) vegetation height (forest vs. low- and medium-height shrubland). Each contrast was sampled using a paired design, with two nearby sites differing in one focal variable while a comparable local landscape context. Within each site pair, the two sites represented contrasting ends of the focal environmental contrast (e.g., forested vs. shrubby, south- vs. north-facing slopes). These contrasts are relative and do not represent fully open or extreme conditions. Each observed site was equipped with three microclimate loggers, resulting in three replicate comparisons per environmental contrast.

**Fig. 1:**
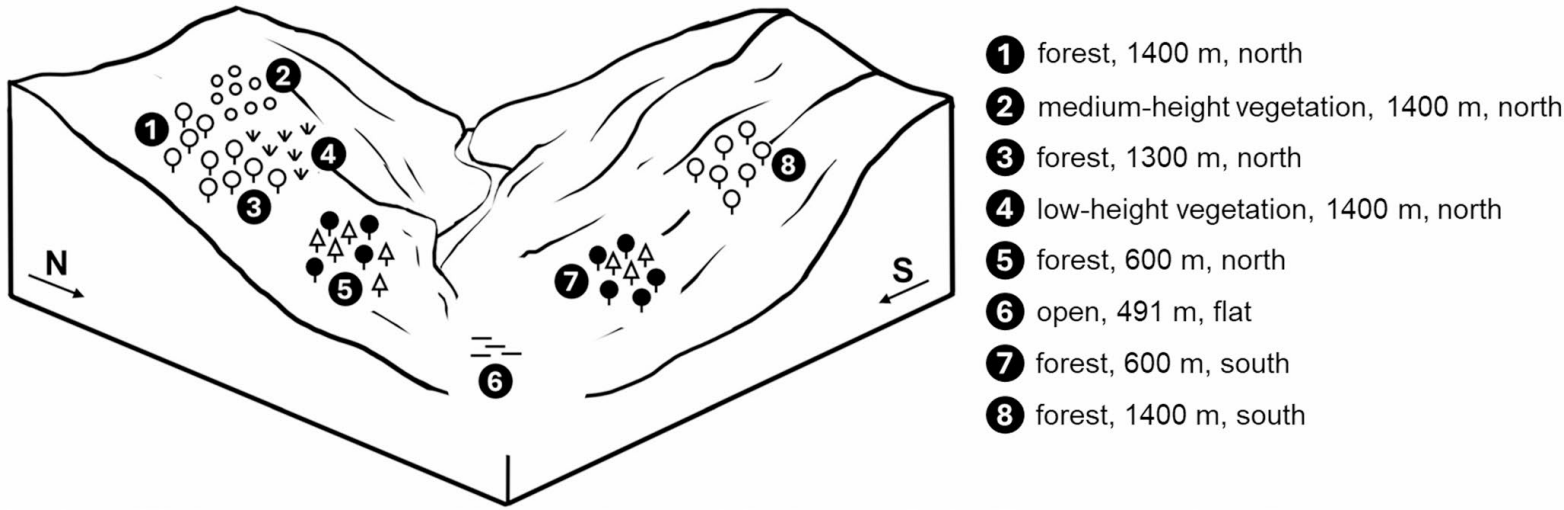
Overview of the study area in the Río Manso Valley showing the locations of microclimate loggers across three environmental contrasts: vegetation height, elevation, and slope orientation. Site numbers correspond to contrasting conditions used in the paired-sampling design (see Table 1)

The spatial layout of the contrasting pairs is shown in Fig. 1 and listed in Table 1. Loggers used for elevation and slope orientation contrasts (sites 1, 5, 7, and 8) were located at 600 m and 1400 m on both north- and south-facing slopes, all within forest stands. At each elevation, six loggers were deployed (three per slope orientation) totalling 12 units that simultaneously captured both environmental contrasts. Forest composition varied with elevation, with low-elevation stands dominated by *Nothofagus dombeyi* and *Austrocedrus chilensis*, and high-elevation sites by *Nothofagus pumilio* and dense *Chusquea culeou* understory. As shown in Table 1, elevation and slope orientation contrasts were structured to allow comparisons within forested conditions while minimizing overlap between environmental contrasts. Elevation was replicated at two slope orientations (north and south), and aspect at two elevations (600 m and 1400 m), thereby reducing overlap among environmental contrasts.

**Table 1:** Overview of paired environmental contrasts used in the study and the corresponding site pairs. Each contrast represents a comparison between two nearby sites differing in a single focal variable (elevation, slope orientation, or vegetation height) within a comparable local landscape context. Site numbers refer to locations shown in Fig. 1

Contrast label	Definition of environmental contrast	Site pairs
E-N	Low vs. high elevation on north-facing slopes	1 and 5
E-S	Low vs. high elevation on south-facing slopes	7 and 8
A-H	North- vs. south-facing slopes at high elevations	1 and 8
A-L	North- vs. south-facing slopes at low elevations	5 and 7
V-M	Medium-height vegetation vs. forest	1 and 2
V-L	Low-height vegetation vs. forest	3 and 4

Vegetation contrasts were restricted to north-facing slopes, where shrubland naturally occurs in the study area, while south-facing slopes are typically forested (Gowda et al. 2012). At 1300 m, three loggers were installed in *Nothofagus pumilio* forest and three in adjacent low shrubland (sites 3 and 4), which featured sparse *Nothofagus antarctica* individuals under 1 m tall and patches of grass and bare ground. At 1400 m, three more loggers were placed in dense medium-height shrubland (1.5–2.5 m, dominated by *Nothofagus antarctica*, site 2) and matched to the adjacent forest loggers (site 1). Logger spacing (10–100 m) was designed to balance local comparability with the ability to capture fine-scale thermal variation.

In addition, one logger was placed at 491 m in an open, flat area (site 6) to provide contextual information on thermal exposure at the valley bottom and was not included in the paired analysis.

### Statistical analysis

Prior to analysis, we imported and pre-processed the temperature time series, including cleaning for missing, duplicated, and temporally misaligned records, and restricted all data to the austral summer months (December–February). To align with the natural diurnal temperature cycle, we computed four daily metrics for each logger: (i) maximum temperature (°C), (ii) minimum temperature (°C), (iii) warming rate (K per hour), and (iv) cooling rate (K per hour). These metrics were selected to characterize different aspects of near-surface thermal dynamics, capturing both peak temperature conditions and the temporal dynamics of warming and cooling at high temporal resolution. The warming rate was calculated from the last minimum temperature preceding the daily maximum, while the cooling rate was based on the subsequent minimum following the maximum. Importantly, the latter was defined as the first minimum occurring between two consecutive daily maxima, rather than being restricted to a fixed calendar day.

$$\:W=\:\frac{{{\rm\:T}}_{max}-\:{T}_{min}^{\leftarrow\:}}{{t}_{max}-\:{t}_{min}^{\leftarrow\:}}$$

$$\:C=\:\frac{{T}_{max}-\:{T}_{min}^{\to\:}}{{t}_{max}-\:{t}_{min}^{\to\:}}$$

Where $\:W$ and $\:C$ are the warming and cooling rates in K/hour, $\:{T}_{max}$ is the maximum temperature of the day in °C, $\:{T}_{min}^{\leftarrow\:}$ is the last minimum temperature before $\:{T}_{max}$ in °C, $\:{T}_{min}^{\to\:}$ is the first minimum temperature after $\:{T}_{max}$, and $\:{t}_{max}$, $\:{t}_{min}^{\leftarrow\:}$, $\:{t}_{min}^{\to\:}$ and are the corresponding timestamps to the measured temperature. Daily maximum and minimum temperatures were identified directly from the 15-minute time series for each logger. To ensure a consistent representation of the diurnal cycle, minimum temperatures were assigned using a shifted temporal window (+ 9 h), allowing early morning minima to be associated with the preceding daytime warming phase. This ensures that warming rates are calculated from the last minimum preceding the daily maximum, while cooling rates are based on the subsequent minimum following the maximum. Because the calculation of warming and cooling rates depends on the identification of distinct diurnal extrema, days with implausible temporal sequences (e.g., minimum temperatures occurring after the daily maximum within the same warming phase) were excluded from the analysis. Such cases were rare and affected only a small number of observations per contrast (see Supplementary Tables 3 and 4), ensuring robustness of the derived metrics under conditions with weak or irregular diurnal temperature cycles. For simplicity, we refer to these variables as “Warming”, “TMAX”, and “Cooling” in the figures. In addition to the main thermal metrics, we performed a supplementary analysis of summer frost exposure. For this, we calculated the number of nights with sub-zero temperatures, using a nighttime window from 20:00 to 10:00 the following day.

Based on the paired sampling design, in which each environmental contrast was represented by three replicate loggers per site, we calculated daily differences in temperature metrics (warming rate, maximum temperature, cooling rate) per pair for each environmental contrast.

$$\:\varDelta\:Y=\:{Y}_{{warmer}_{i}}-\:{Y}_{{cooler}_{i}}$$

Where $\:Y$ represents $\:W$, $\:{T}_{max}$, or $\:C$ and $\:i$ is the $i-th$ pair. This resulted in three $\:\varDelta\:Y$ values per day and contrast, reflecting replicate-level thermal differences. For each environmental contrast, site $\:A$ was defined as the end of the contrast typically associated with higher summer temperatures (i.e. forest vs. shrubland, high vs. low elevation, and south- vs. north-facing slopes), while site $\:B$ represented the opposite end.

These daily differences were then used as response variables in linear mixed-effects models, fitted using the *nlme* package in RStudio (Pinheiro and Bates 2025). For each contrast, we expected approximately 270 replicate-level differences in temperature and rate metrics (based on three paired replicates across 90 summer days), with each value representing the difference between the two sites forming a paired contrast.

To assess how thermal differences varied across environmental contrasts and with temperature, we implemented a two-step modelling approach. In the first step, we fitted linear mixed-effects models without temperature covariates to estimate the average differences in thermal metrics across each environmental contrast during the summer period. These models provide a baseline description of contrast-specific differences in thermal metrics under typical summer conditions. In the second step, we included the maximum temperature at the warmer site of each paired contrast $\:{T}_{max}^{w}$ as a covariate to test whether thermal differences varied with increasing summer temperature. This allowed us to assess whether contrast-specific differences became more pronounced as temperatures increase.

Because the environmental contrasts were intentionally sampled as paired comparisons within specific landscape contexts (e.g., vegetation contrasts restricted to north-facing slopes and elevation contrasts conducted within forest stands), linear mixed-effects models were fitted separately for each contrast and thermal metric. This contrast-wise modelling approach preserves the paired structure of the data and ensures that estimates are derived within comparable environmental contexts, rather than being mixed across contrasts. As a result, effect sizes are not directly comparable among vegetation, elevation, and slope orientation within a single unified model, and differences in their relative importance are evaluated across contrasts rather than through formal statistical comparison. Within each model, to account for residual variability between replicate sites (e.g., microsite-level differences) and temporal dependence in the daily data, we included a random intercept for replicate identity and an autoregressive correlation structure (AR(1)). Model assumptions, including residual normality, homoscedasticity, and autocorrelation, were checked using diagnostic plots and were met within acceptable limits.

The model structure without covariate was:

$$\:\varDelta\:Y\:\sim\:1+\left(1\:\right|\:replicate)+AR(1)$$

The model structure with the covariate was:

$$\:\varDelta\:Y\:\sim\:{T}_{max}^{w}+\left(1\:\right|\:replicate)+AR(1)$$

where $\:\varDelta\:Y$ represents the daily difference in maximum temperature, warming slope, and cooling slope, respectively.

To describe the variance explained by the models, we calculated marginal and conditional R² following the framework proposed by Nakagawa and Schielzeth (2013) and extended by Johnson (2014). These metrics were computed using the *MuMIn* package in RStudio (Bartoń 2025). Model assumptions (normality of residuals, homoscedasticity, temporal structure) were validated using diagnostic plots and were met within acceptable limits.

## Results

### Observed thermal patterns and microclimatic differences across environmental contrasts

A descriptive analysis of the observed temperature data (Supplementary Table 1) reveals that the mean daily maximum temperature across all environmental contrasts was 25.2 °C (IQR = 8.0). The highest mean value occurred at the low-elevation, north-facing slope (see Fig. 1, site 5), reaching 30.8 °C (IQR = 9.4). For comparison, the mean temperature at the reference site (see Fig. 1, site 6) was 32.3 °C (IQR = 8.5), indicating that near-surface temperatures at the study sites remained consistently lower than those recorded at the valley-bottom reference location.

The average time of day when maximum temperatures occurred across all environmental contrasts was 15:00 (IQR = 1.75 h). The earliest median time of daily maximum temperature was observed in the medium-height vegetation (see Fig. 1, site 2) at 13:30 (IQR = 1.5 h), while the latest occurred at high elevations on south-facing slopes (see Fig. 1, site 8) at 16:00 (IQR = 2.5 h). At the reference site, the median time of maximum temperature was 14:30 (IQR = 2 h).

In addition to daytime temperature extremes, site-averaged temperature records revealed occasional nighttime frost events during the austral summer (see Supplementary Table 2). The highest number of frost nights was recorded at the open valley-bottom reference site (see Fig. 1, site 6), with 38 frost nights. Shrubland sites experienced 7 to 9 frost nights, while forest sites ranged from 0 to 9, depending on elevation and aspect.

**Fig. 2:**
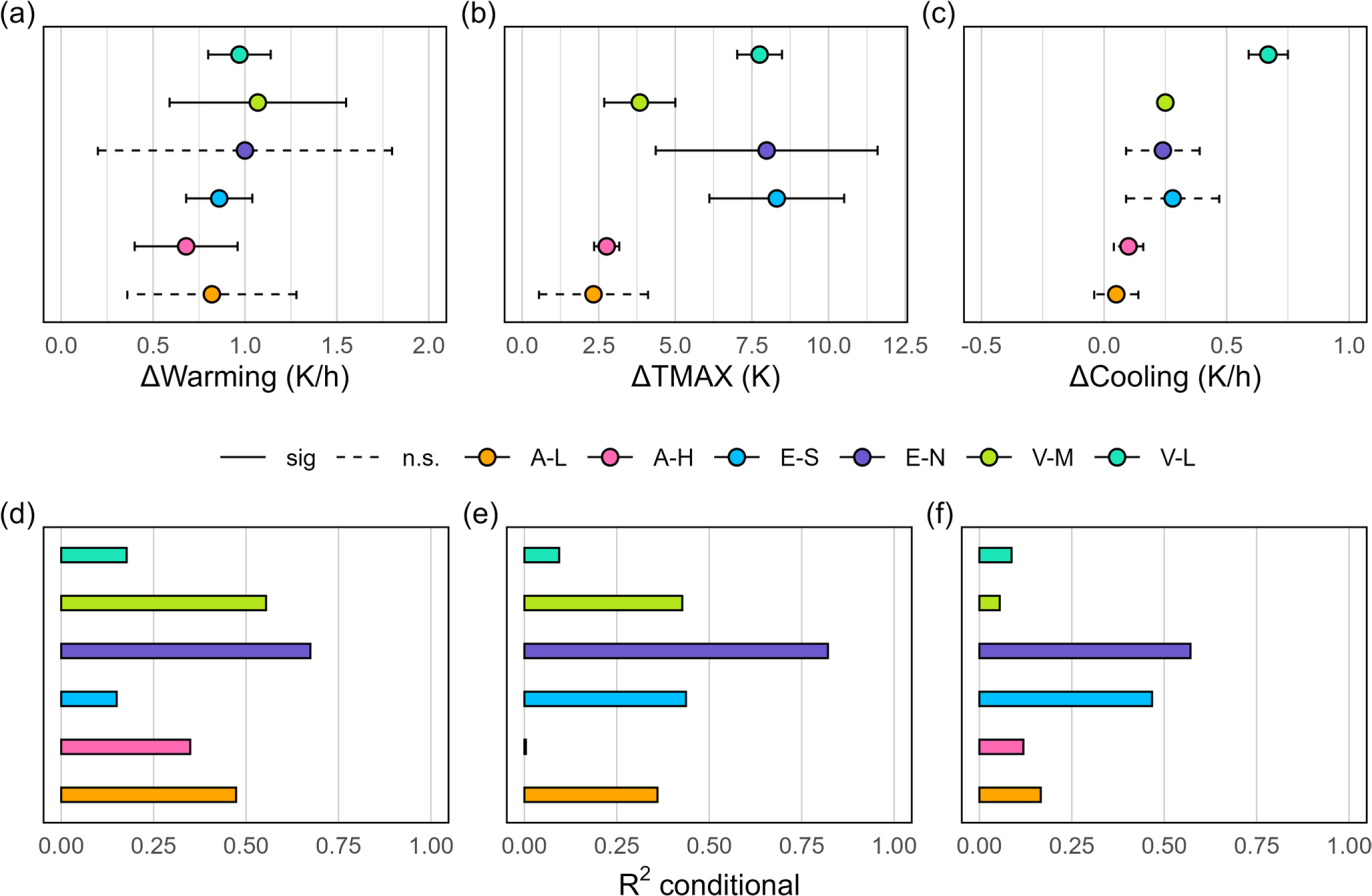
Daily differences in (**a**, **d**) warming rate (K/h), (**b**, **e**) maximum temperature (K), and (**c**, **f**) cooling rate (K/h) between paired sites across six environmental contrasts: vegetation height (low-height [V-L], medium-height [V-M]), elevation (north-facing [E-N], south-facing [E-S]), and slope orientation (high elevation [A-H], low elevation [A-L]). Panels (**a**–**c**) show marginal means (± 95% confidence intervals) estimated from linear mixed-effects models fitted with an autoregressive correlation structure. Solid lines indicate significant differences (*p* < 0.05); dashed lines indicate non-significant results. Panels (**d**–**f**) display conditional R² values for each model, reflecting the proportion of variance associated with both fixed and random effects

In models without covariates, differences in warming rate, maximal temperature, and cooling rate varied substantially across environmental contrasts (Fig. 2).

For the warming rate differences (Fig. 2 panel a and d; Supplementary Table 3), the environmental contrasts V-L (0.97 K/h, *p* < 0.001), V-M (1.07 K/h, *p* < 0.05), E-S (0.86 K/h, *p* < 0.001), and A-H (0.68 K/h, *p* < 0.05) showed significant differences, whereas contrasts E-N (1.00 K/h, *p* = 0.213) and A-L (0.82 K/h, *p* = 0.075) did not. Conditional R² values varied among contrasts, ranging from 0.15 (E-S) to 0.55 (V-M), reflecting differing contributions of fixed and random effects across models.

For maximum temperature differences (Fig. 2 panel b and e; Supplementary Table 3), the largest effects were observed for elevation contrasts, particularly on south-facing slopes (E-S: 8.31 K) and north-facing slopes (E-N: 7.98 K), both significant (*p* < 0.001 and *p* < 0.05). The low-height vegetation contrast (V-L) also showed a large and significant difference (7.75 K, *p* < 0.001). In contrast, the medium-height vegetation contrast (V-M: 3.84 K) and slope orientation contrasts (A-H: 2.76 K, A-L: 2.33 K) exhibited smaller differences, with A-L not reaching significance (*p* = 0.192). Conditional R² values varied widely among contrasts, ranging from 0.004 (A-H) to 0.82 (E-N).

Cooling rate differences (Fig. 2 panel c and f; Supplementary Table 3) were generally smaller. Significant differences were detected for both vegetation contrasts: V-L (0.67 K/h, *p* < 0.001) and V-M (0.25 K/h, *p* < 0.001), whereas all other contrasts showed lower effect sizes and were not significant (*p* > 0.1). Conditional R² values ranged from 0.06 (V-M) to 0.57 (E-N).

### Temperature dependence of microclimatic differences across environmental contrasts

**Fig. 3:**
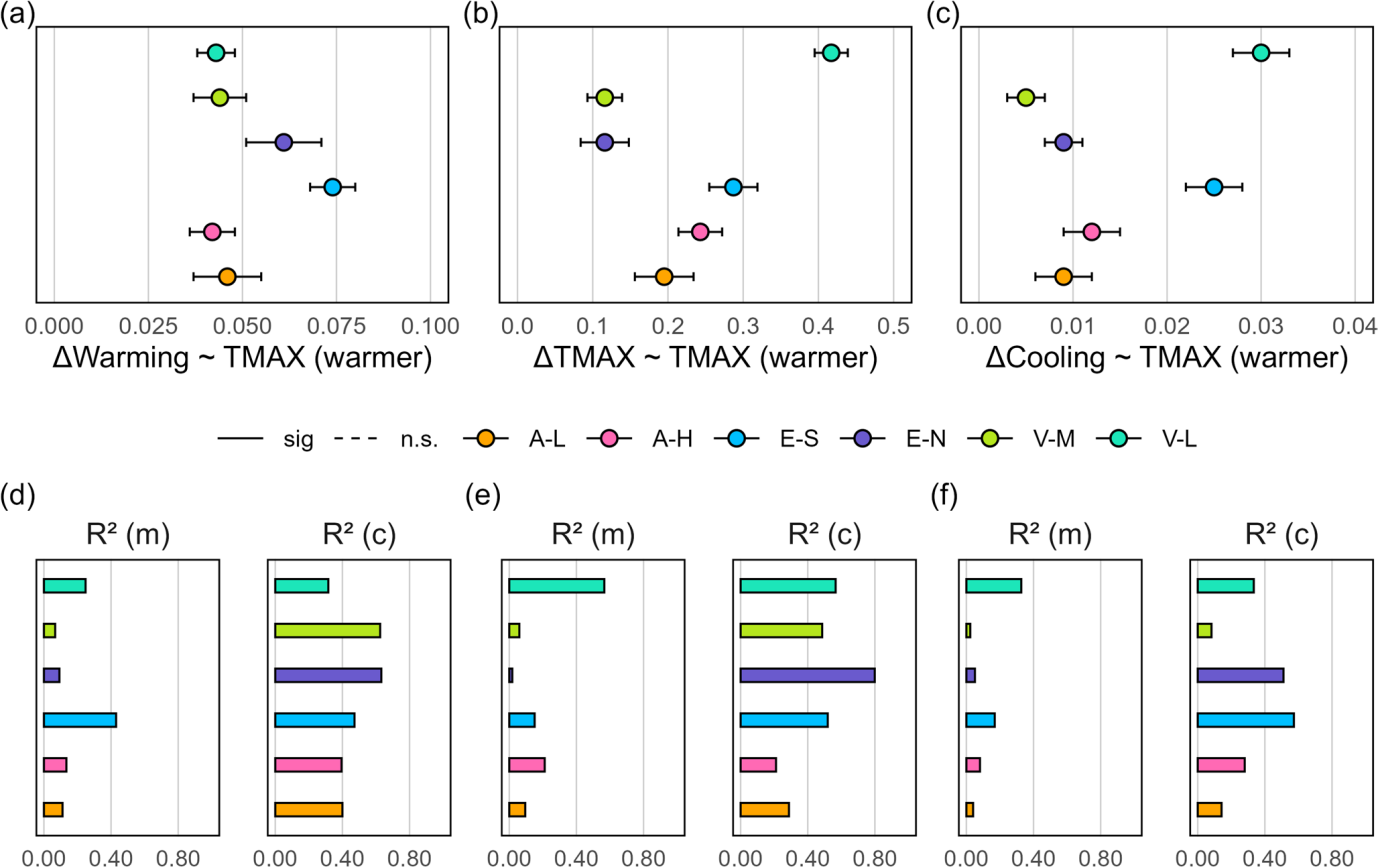
Temperature dependence of microclimatic differences in (**a**, **d**) warming rate (K/h), (**b**, **e**) maximum temperature (K), and (**c**, **f**) cooling rate (K/h) across six environmental contrasts: vegetation height (low-height [V-L], medium-height [V-M]), elevation (north-facing [E-N], south-facing [E-S]), and slope orientation (high elevation [A-H], low elevation [A-L]). Panels (**a**–**c**) show model-estimated slopes (± 95% confidence intervals) from linear mixed-effects models including daily maximum temperature at the warmer site of each paired contrast as a covariate. Slopes describe how differences in thermal metrics vary with increasing summer temperatures. Solid lines indicate significant relationships (*p* < 0.05); dashed lines indicate non-significant relationships. Panels (**d**–**f**) display marginal (left) and conditional (right) R² values for each model

When daily maximum temperature at the warmer site of each paired contrast was included as a covariate, differences in warming rate, maximum temperature, and cooling rate showed a significant temperature dependence across all environmental contrasts (Fig. 3). For the medium-height vegetation (V-M) and low-elevation aspect (A-L) contrasts, significance level reached *p* < 0.05 and *p* < 0.005, respectively, whereas all other contrasts consistently exhibited stronger temperature-dependent effects (*p* < 0.001).

For warming rate differences (Fig. 3 panel a and d, Supplementary Table 4), all environmental contrasts showed significant positive relationships with daily maximum temperature at the warmer site of each paired contrast. Estimated slopes ranged from 0.042 (A-H) to 0.074 (E-S), with the largest proportion of variance explained by fixed effects observed for the elevation contrast on south-facing slopes (E-S: marginal R² = 0.43), followed by the low-height vegetation contrast (V-L: marginal R² = 0.25). Medium-height vegetation (V-M), low-elevation aspect (A-L), and north-facing elevation (E-N) contrasts showed weaker associations (marginal R² between 0.07 and 0.11), whereas conditional R² values were generally higher across contrasts, reflecting the contribution of random effects.

For maximum temperature differences (Fig. 3 panel b and e; Supplementary Table 4), all environmental contrasts showed significant and positive relationships with daily maximum temperature at the warmer site of each paired contrast. Estimated slopes were largest for the low-height vegetation contrast (V-L: 0.417), followed by the elevation contrast on south-facing slopes (E-S: 0.287), the high-elevation aspect contrast (A-H: 0.243), and the low-elevation aspect contrast (A-L: 0.195). Weaker but still significant relationships were observed for the medium-height vegetation (V-M) and north-facing elevation (E-N) contrasts (both 0.116).

The proportion of variance explained by fixed effects varied substantially among contrasts. The low-height vegetation contrast (V-L) showed the highest marginal R² (0.57), whereas the north-facing elevation contrast (E-N) exhibited a low marginal R² (0.02) but a high conditional R² (0.80), indicating that a large share of the variation was associated with random effects rather than with temperature dependence alone.

Cooling rate differences (Fig. 3 panel c and f; Supplementary Table 4) also showed significant positive relationships with daily maximum temperature across all environmental contrasts. Estimated slopes were consistently smaller than those observed for warming rate differences, ranging from 0.005 for the medium-height vegetation contrast (V-M) to 0.030 for the low-height vegetation contrast (V-L). The low-height vegetation contrast (V-L) exhibited both the largest slope (0.030) and the highest marginal R² (0.33), whereas other contrasts showed weaker temperature-dependent relationships, with marginal R² values mostly below 0.17.

**Fig. 4:**
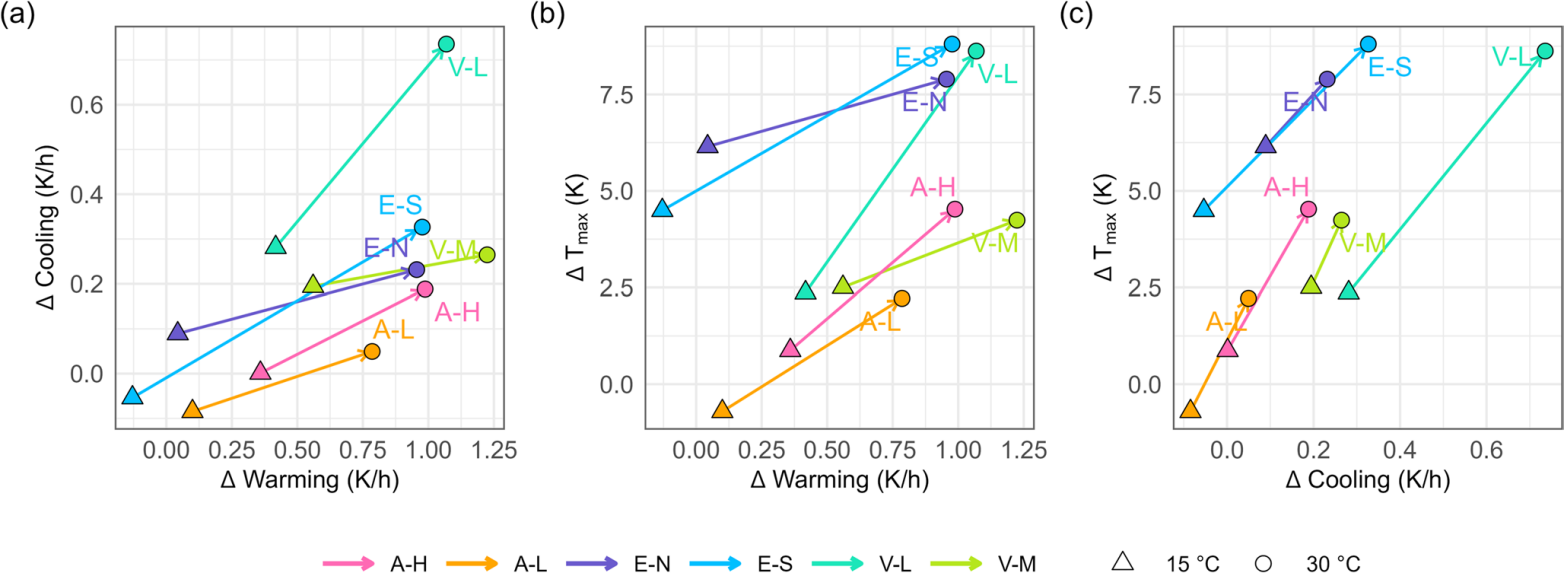
Model-predicted differences in warming rate, cooling rate, and maximum temperature across six environmental contrasts at daily maximum temperatures of 15 °C and 30 °C. Arrows connect predictions at 15 °C (base) and 30 °C (tip), illustrating how microclimatic differences between paired sites change with increasing temperature (see Fig. 3)

To illustrate how microclimatic differences varied across temperature regimes, we compared model-predicted differences at 15 °C and 30 °C for all three metrics across the six environmental contrasts (Fig. 4). Across all contrasts, differences increased between 15 °C and 30 °C, indicating a consistent amplification of thermal contrasts under warmer conditions. However, the magnitude of this increase varied among contrasts. The low-height vegetation contrast (V-L) showed the largest and most consistent increases in predicted differences at 30 °C, distinguishing it from most other contrasts across metrics. Elevation contrasts exhibited pronounced changes in warming rate differences, particularly on south-facing slopes (E-S), whereas warming rate differences were more evident for the medium-height vegetation contrast (V-M). These results indicate that the magnitude of microclimatic differences varies across environmental contrasts and temperature levels. These results highlight that both the strength and the temperature sensitivity of microclimatic differences depend on the type of environmental contrast.

## Discussion

### Differences in near-surface thermal responses across vegetation, elevation, and slope orientation

Our findings show that near-surface microclimatic differences are context-dependent and vary with temperature (Davis et al. 2019; De Frenne et al. 2019; Starck et al. 2025). Across paired environmental contrasts, the magnitude and statistical support for differences in warming rate, maximum temperature, and cooling rate varied among contrast types and thermal metrics. Together, these patterns indicate that summer thermal sensitivity differs among vegetation, elevation, and slope-orientation contrasts and becomes more pronounced at higher temperatures.

Vegetation contrasts showed the largest and most consistent differences in near-surface thermal metrics. Forest stands dominated by *Nothofagus pumilio* exhibited lower maximum temperatures and smaller warming and cooling rates than adjacent low-height shrubland. This pattern aligns with studies showing that forest canopies and associated structure can moderate near-surface thermal extremes and diurnal dynamics (Jucker et al. 2018; Díaz-Calafat et al. 2023; Csölleová et al. 2024; John et al. 2024), and is further supported by regional observations from Simon et al. (2024), who reported pronounced temperature differences between forested sites and more open forest conditions. This pattern is consistent with established mechanisms in which vegetation structure controls the surface energy balance. In forested sites, reduced shortwave radiation input due to canopy shading limits daytime energy accumulation, while higher evapotranspiration promotes latent heat flux and constrains near-surface warming. In contrast, structurally open shrubland sites receive greater direct solar radiation, resulting in stronger daytime heating and larger thermal amplitudes (De Frenne et al. 2019, 2021). The observed increase in warming rates and maximum temperature differences under higher temperature conditions is consistent with increasing energy input amplifying these processes, leading to stronger divergence in thermal dynamics between vegetation types during warm periods. While both low-height (< 1 m) and medium-height (1.5–2.5 m) vegetation contrasts differed significantly from forest sites, the magnitude and temperature dependence of these differences varied, although these patterns should be interpreted in the context of the study design, as vegetation contrasts were restricted to north-facing slopes, which are typically warmer and more exposed, and may therefore amplify the observed differences between forest and shrubland sites. Low-height vegetation showed consistently large differences in warming rate, maximum temperature, and cooling rate, with differences increasing under warmer conditions. In contrast, medium-height vegetation showed smaller differences and weaker temperature dependence, particularly for cooling rate and maximum temperature. This supports findings from post-fire landscapes where sparse or low-stature vegetation allows intense daytime heating and limited nocturnal retention (Marsh et al. 2022a, b). Even modest increases in vegetation height have been shown to reduce thermal variability, consistent with vegetation-microclimatic feedbacks (D’Odorico et al. 2010, 2013). Notably, the frequency of summer frost nights further illustrates differences in near-surface thermal conditions among vegetation contrasts: Low-height vegetation experienced nine frost nights, compared to just one at its paired forest site. In contrast, medium-height vegetation recorded seven frost nights, slightly fewer than its adjacent forest site, which recorded nine. The open valley-bottom reference site showed the highest number of frost nights (38). While these patterns are consistent with stronger nocturnal cooling in structurally open sites, cold air drainage and accumulation in lower-lying terrain may also contribute to frost occurrence. However, the presence of forested sites at lower elevations with comparatively fewer frost events suggests that vegetation structure plays an additional role in modulating nocturnal temperature dynamics. As such, the observed patterns likely reflect interacting effects of vegetation and topographic position that cannot be fully disentangled within the present study design.

Topographic contrasts showed more complex and less consistent patterns of near-surface thermal differences. These patterns are consistent with differences in incoming solar radiation and surface energy balance associated with slope orientation and elevation (Geiger et al. 2009; Dobrowski 2011). In the elevation contrasts, south-facing slopes exhibited both large differences in maximum temperature and strong temperature dependence. In contrast, north-facing slopes also exhibited large temperature differences but much weaker relationships with maximum temperature. This asymmetry suggests that elevation interacts with slope orientation, such that temperature dependence differs between north- and south-facing slopes. Under these conditions, stronger radiation input on north-facing slopes may lead to consistently high energy availability, limiting additional temperature-dependent divergence, whereas on south-facing slopes, lower baseline radiation may allow differences to increase more strongly at higher temperatures (Barry 2008). These findings indicate that elevation effects depend on slope orientation and broader landscape context, rather than acting independently (Geiger et al. 2009; Dobrowski 2011). Slope orientation contrasts showed smaller and more variable near-surface thermal differences than vegetation and elevation contrasts. In both slope-orientation contrasts, differences in maximum temperature were moderate in magnitude, with only the high-elevation contrast showing significant differences. The slope-orientation contrast at high elevation also showed a significant increase in temperature differences with rising daily maximum temperature. Together, these results suggest that slope orientation exerts a weaker and more context-dependent influence on near-surface thermal conditions than vegetation or elevation, with clearer effects emerging primarily at high elevations and higher temperatures.

Overall, these results show that near-surface thermal differences vary systematically across environmental contrasts and temperature conditions. Vegetation, elevation, and slope orientation jointly shape near-surface temperature regimes through their influence on radiation input and surface energy exchange. The increase in warming rates and maximum temperature differences at higher temperatures is consistent with greater solar radiation and energy input amplifying these processes, leading to stronger divergence in thermal dynamics between vegetation types during warm periods. As illustrated in Fig. 4, this amplification is not uniform across contrasts. Vegetation contrasts show the strongest increase between moderate and high temperature conditions, whereas slope orientation contrasts exhibit weaker and more variable responses. This highlights that the sensitivity of near-surface thermal dynamics to warming depends on how strongly local conditions modify energy inputs and exchanges. However, given that spatial replication was limited to three replicate loggers per site pair, the observed patterns primarily reflect microclimatic dynamics within the Río Manso Valley, and the scope for broader generalization beyond this landscape context remains constrained. The implications should therefore be interpreted as hypotheses and mechanisms that remain to be tested across a broader range of environmental settings. Within this context, these insights are directly relevant for improving forest microclimate models developed for the same study area, such as those presented by Fierke et al. (2025), and may also inform applications in comparable temperate mountain systems, where fine-scale effects of vegetation characteristics and topography are often underrepresented. By quantifying fine-scale thermal variation across vegetation and topographic contrasts, our study helps refine spatial predictions of these interacting dynamics under climate change within similar landscape contexts.

### Potential implications for microclimate-sensitive processes in fire-prone landscapes

Our findings provide a framework for understanding how near-surface microclimates mediate key ecological processes in fire-prone landscapes, particularly forest regeneration and surface flammability. Seedling establishment is highly sensitive to microsite conditions, with near-surface temperature dynamics influencing germination, dehydration risk, and early growth (Hill et al. 2024; Espinosa del Alba et al. 2025). Forested sites, which showed consistently lower maximum temperatures and reduced diurnal temperature variability relative to adjacent open sites, may function as microrefugia for early life stages by protecting seedlings from increasingly frequent heat extremes (von Arx et al. 2013). In contrast, shrubland sites showed pronounced diurnal temperature fluctuations, combining high daytime temperatures with rapid nocturnal cooling. These regimes may expose seedlings to multiple stressors, potentially favouring fast-growing, heat-tolerant species, while limiting recruitment of shade- or drought-sensitive taxa and altering successional dynamics. At the same time, shrublands and north-facing slopes at low elevation may experience conditions conducive to surface fuel drying, which has been linked to increased ignition probability and fire spread during hot and dry conditions (Blackhall et al. 2017; Barberá et al. 2023). These patterns are consistent with fire probability models in northwestern Patagonia, which identify the highest burn risk at low elevations with intermediate precipitation and flammable vegetation such as shrublands (Barberá et al. 2025). While Barberá et al. (2025) identified statistical relationships between fire occurrence, vegetation, and environmental factors, our results add fine-scale microclimatic context by resolving near-surface temperature dynamics across contrasting vegetation and topographic settings. On north-facing slopes, open vegetation was associated with higher near-surface temperatures and more rapid diurnal temperature changes, conditions that are linked to enhanced surface fuel drying. Although our vegetation contrasts were limited to north-facing slopes and therefore cannot fully disentangle vegetation effects from slope-related influences, the observed patterns suggest that vegetation characteristics may contribute to shaping near-surface thermal conditions relevant to fire behaviour within the study landscape. Moreover, recurrent fires in these environments may reinforce thermal extremes by reducing forest cover and promoting shrub encroachment, creating feedbacks that sustain flammable landscape states (Paritsis et al. 2015; Tiribelli et al. 2018). These coupled patterns illustrate how near-surface microclimate can influence both regeneration dynamics and disturbance risk, and how each process may reinforce the other. By quantifying fine-scale thermal variation across vegetation and topographic contrasts, our study helps refine spatial predictions of these interacting dynamics under climate change within similar landscape contexts. While the observed thermal patterns provide plausible links to regeneration dynamics and fire risk, their broader ecological significance remains to be tested beyond the specific environmental context represented in this study.

### Limitations and future directions

While our study provides detailed insights into near-surface thermal differences across vegetation, elevation, and slope orientation, several limitations should be acknowledged. First, the independence of environmental contrasts is partly constrained by the study design, as vegetation contrasts were exclusively sampled on north-facing slopes. As a result, vegetation effects cannot be fully disentangled from potential influences of slope orientation, and some of the observed differences may reflect combined effects of vegetation structure and aspect. Second, spatial replication was limited to three replicate loggers per site pair, meaning that much of the statistical power arises from temporal replication rather than fully independent spatial sampling, which constrains the extent to which these findings can be generalized beyond the study area. Third, beyond these design constraints, our sensor network captures fine-scale near-surface temperature variation but does not include other variables such as humidity, wind, or radiation, which are also known to influence near-surface thermal conditions (Davis et al. 2019). Fourth, our design focuses on binary contrasts (e.g., forest vs. low- and medium-height vegetation), which limits our ability to assess microclimatic variation along continuous structural or compositional transitions. This approach may overlook gradual vegetation changes and edge effects that occur across finer-scale transitions (Meeussen et al. 2021). Finally, although we recorded a full year of data including multiple heat events, longer-term monitoring will be needed to assess interannual variability and cumulative effects, particularly in relation to large-scale climate drivers such as the El Niño–Southern Oscillation and the Southern Annular Mode.

Future studies should integrate parameters such as soil moisture, humidity, wind, and radiation to allow a more complete evaluation of near-surface thermal variation and temperature dependent differences. The use of remote sensing, including LiDAR-based vegetation metrics, could help quantify forest structural complexity and extend the spatial scope of these findings. Linking microclimate data to nearby weather stations would facilitate upscaling and integration with regional climate models. Overall, this study provides a first step toward a broader framework for understanding temperature-sensitive microclimatic variation and its ecological implications in forest ecosystems of northern Patagonia.

## Supplementary information

Below is the link to the electronic supplementary material.

Supplementary File 1 (DOCX 42.1 KB)

## Data Availability

Derived data supporting the findings of this study are available upon request from the corresponding author.
