# Supplementary material for: Contrasting vegetation height and elevation shape near-surface thermal responses more strongly than slope orientation during summer in northern Patagonia
Source: Int J Biometeorol. 2026 Jun 29;70(7):197. doi: 10.1007/s00484-026-03254-w (PMC13315477; doi:10.1007/s00484-026-03254-w)
Supplement: Supplementary file 1 — (DOCX 42.1 KB) [file 484_2026_3254_MOESM1_ESM.docx]

**Article title:** Contrasting vegetation height and elevation shape near-surface thermal responses more strongly than slope orientation during summer in northern Patagonia

**Journal name:** International Journal of Biometeorology

**Author names:** Jonas Fierke, Juan Haridas Gowda, Gastón Mauro Díaz, Philipp Koal, Helge Walentowski, Martin Kappas, Birgitta Putzenlechner

**Affiliation and e-mail address of the corresponding author:** University of Göttingen, Institute of Geography, Goldschmidtstraße 3, 37077 Göttingen, Germany (jonas.fierke@uni-goettingen.de)

**Supplementary information**

**Table 1.** Summary statistics of temperature and diurnal metrics recorded at eight study sites in the Río Manso Valley during the austral summer. Variables include daily maximum temperature (TMAX), minimum temperature (TMIN), and their respective times of occurrence (TMAX Time, TMIN Time), as well as warming and cooling rates. For each variable and site, sample size (n), median, interquartile range (IQR), minimum (Min), and maximum (Max) are reported. Time values are presented in HH:MM format.

| **Variable** | **Site** | **n** | **Median** | **IQR** | **Min** | **Max** |
| --- | --- | --- | --- | --- | --- | --- |
| **TMAX** | 1 | 264 | 23.16 | 7.56 | 6.63 | 39.75 |
|  | 2 | 264 | 27.28 | 8.48 | 7.50 | 39.94 |
|  | 3 | 264 | 20.63 | 7.30 | 6.19 | 34.25 |
|  | 4 | 265 | 28.50 | 9.44 | 6.63 | 44.00 |
|  | 5 | 268 | 30.75 | 9.41 | 10.75 | 51.38 |
|  | 6 | 90 | 32.28 | 8.47 | 17.38 | 41.50 |
|  | 7 | 264 | 28.56 | 8.92 | 12.44 | 46.81 |
|  | 8 | 263 | 20.19 | 7.50 | 6.75 | 34.69 |
| **TMIN** | 1 | 264 | 5.81 | 5.28 | -1.88 | 13.38 |
|  | 2 | 264 | 4.84 | 5.58 | -2.25 | 13.13 |
|  | 3 | 264 | 6.16 | 5.25 | -1.13 | 13.81 |
|  | 4 | 265 | 4.56 | 5.75 | -3.38 | 13.75 |
|  | 5 | 268 | 9.31 | 4.33 | 2.75 | 16.88 |
|  | 6 | 90 | 1.19 | 8.61 | -5.38 | 24.63 |
|  | 7 | 264 | 9.00 | 4.52 | 2.00 | 17.13 |
|  | 8 | 263 | 6.38 | 5.47 | -1.56 | 14.88 |
| **TMAX Time** | 1 | 264 | 14:30 | 1:30 | 10:00 | 17:15 |
|  | 2 | 264 | 13:30 | 1:30 h | 10:45 | 17:15 |
|  | 3 | 264 | 15:15 | 2:00 h | 10:45 | 19:30 |
|  | 4 | 265 | 15:30 | 2:00 h | 10:30 | 19:30 |
|  | 5 | 268 | 14:15 | 2:15 h | 10:30 | 17:30 |
|  | 6 | 90 | 14:30 | 2:00 h | 0:00 | 22:00 |
|  | 7 | 264 | 15:30 | 0:45 h | 12:30 | 19:00 |
|  | 8 | 263 | 16:00 | 2:30 h | 10:15 | 19:30 |
| **TMIN Time** | 1 | 264 | 06:30 | 01:15 h | 02:00 | 08:15 |
|  | 2 | 264 | 06:15 | 01:15 h | 01:30 | 08:30 |
|  | 3 | 264 | 06:30 | 01:15 h | 02:00 | 09:15 |
|  | 4 | 265 | 06:15 | 01:30 h | 00:30 | 09:00 |
|  | 5 | 268 | 07:15 | 00:45 h | 00:30 | 09:30 |
|  | 6 | 90 | 06:30 | 01:15 h | 00:00 | 22:45 |
|  | 7 | 264 | 07:00 | 01:00 h | 02:00 | 09:30 |
|  | 8 | 263 | 06:15 | 01:37 h | 01:15 | 09:30 |
| **WARMING** | 1 | 264 | 1.97 | 1.05 | 0.35 | 4.77 |
|  | 2 | 264 | 3.18 | 1.30 | 0.47 | 5.91 |
|  | 3 | 264 | 1.59 | 0.61 | 0.30 | 3.72 |
|  | 4 | 265 | 2.65 | 1.42 | 0.38 | 5.00 |
|  | 5 | 268 | 2.85 | 1.63 | 0.61 | 7.56 |
|  | 6 | 90 | 3.70 | 2.14 | 0.57 | 6.28 |
|  | 7 | 264 | 2.36 | 1.22 | 0.34 | 4.12 |
|  | 8 | 263 | 1.38 | 0.59 | 0.24 | 3.50 |
| **COOLING** | 1 | 264 | 1.07 | 0.37 | 0.19 | 2.00 |
|  | 2 | 264 | 1.33 | 0.35 | 0.39 | 2.06 |
|  | 3 | 264 | 0.92 | 0.30 | 0.18 | 2.24 |
|  | 4 | 265 | 1.57 | 0.61 | 0.21 | 3.44 |
|  | 5 | 268 | 1.27 | 0.54 | 0.25 | 2.60 |
|  | 6 | 90 | 2.02 | 0.87 | 0.20 | 3.26 |
|  | 7 | 264 | 1.20 | 0.54 | 0.21 | 2.64 |
|  | 8 | 263 | 0.93 | 0.40 | 0.16 | 2.04 |

**Table 2.** Number of summer frost nights (nights with at least one 15-minute interval below 0 °C) recorded at each study site during the period from 15 February 2024 to 13 February 2025, based on site-averaged temperature data. Frost nights were identified using nighttime temperature data (20:00–10:00), and sites are grouped by vegetation height, elevation, and slope orientation as described in Figure 1.

| **Site** | **Description** | **Frost nights (n)** |
| --- | --- | --- |
| 1 | Forest, 1400 m, north | 9 |
| 2 | Medium-height vegetation, 1400 m, north | 7 |
| 3 | Forest, 1300 m, north | 1 |
| 4 | Low-height vegetation, 1300 m, north | 9 |
| 5 | Forest, 600 m, north | 0 |
| 6 | Open site, 491 m, flat | 38 |
| 7 | Forest, 600 m, south | 0 |
| 8 | Forest, 1400 m, south | 8 |

**Table 3.** Results of linear mixed-effects models quantifying absolute differences in warming rate (K/h), maximum temperature (K), and cooling rate (K/h) across six environmental contrasts: vegetation height (low-height [V-L], medium-height [V-M]), elevation (north-facing [E-N], south-facing [E-S]), and slope orientation (high elevation [A-H], low elevation [A-L]). For each variable and contrast, model estimates (intercept), standard error (SE), t-value, and p-value are shown. Marginal (R² m) and conditional (R² c) R² values indicate the variance explained by fixed effects alone and by the full model including random effects, respectively.

| **Variable** | **Contrast** | **n (days)** | **Intercept** | **SE** | **t-value** | **p-value** | **R² (m)** | **R² (c)** |
| --- | --- | --- | --- | --- | --- | --- | --- | --- |
| **Warming** | V-L | 264 | 0.97 | 0.17 | 5.68 | <0.001 | 0 | 0.18 |
|  | V-M | 264 | 1.07 | 0.48 | 2.24 | 0.0262 | 0 | 0.55 |
|  | E-N | 264 | 1.00 | 0.80 | 1.25 | 0.2128 | 0 | 0.67 |
|  | E-S | 263 | 0.86 | 0.18 | 4.81 | <0.001 | 0 | 0.15 |
|  | A-H | 263 | 0.68 | 0.28 | 2.44 | 0.0154 | 0 | 0.35 |
|  | A-L | 263 | 0.82 | 0.46 | 1.79 | 0.0750 | 0 | 0.47 |
| **TMAX** | V-L | 264 | 7.75 | 0.73 | 10.64 | <0.001 | 0 | 0.09 |
|  | V-M | 264 | 3.84 | 1.16 | 3.31 | 0.0011 | 0 | 0.43 |
|  | E-N | 264 | 7.98 | 3.62 | 2.20 | 0.0286 | 0 | 0.82 |
|  | E-S | 263 | 8.31 | 2.20 | 3.77 | <0.001 | 0 | 0.44 |
|  | A-H | 263 | 2.76 | 0.41 | 6.70 | <0.001 | 0 | 0.00 |
|  | A-L | 263 | 2.33 | 1.78 | 1.31 | 0.1920 | 0 | 0.36 |
| **Cooling** | V-L | 264 | 0.67 | 0.08 | 8.78 | <0.001 | 0 | 0.09 |
|  | V-M | 264 | 0.25 | 0.03 | 7.53 | <0.001 | 0 | 0.06 |
|  | E-N | 264 | 0.24 | 0.15 | 1.59 | 0.1138 | 0 | 0.57 |
|  | E-S | 263 | 0.28 | 0.19 | 1.50 | 0.1350 | 0 | 0.47 |
|  | A-H | 263 | 0.10 | 0.06 | 1.64 | 0.1028 | 0 | 0.12 |
|  | A-L | 263 | 0.05 | 0.09 | 0.64 | 0.5214 | 0 | 0.17 |

**Table 4.** Results of linear mixed-effects models including maximum temperature at the warmer site as a covariate. Models quantify absolute differences in warming rate (K/h), maximum temperature (K), and cooling rate (K/h) across six environmental contrasts: vegetation height (low-height [V-L], medium-height [V-M]), elevation (north-facing [E-N], south-facing [E-S]), and slope orientation (high elevation [A-H], low elevation [A-L]). For each variable and contrast, model estimates (intercept and slope), standard error (SE), t-value, and p-value are shown. Marginal (R² m) and conditional (R² c) R² values indicate the variance explained by fixed effects alone and by the full model including random effects, respectively.

| **Variable** | **Contrast** | **n (days)** | **Intercept** | **Slope** | **SE** | **t-value** | **p-value** | **R² (m)** | **R² (c)** |
| --- | --- | --- | --- | --- | --- | --- | --- | --- | --- |
| **Warming** | V-L | 264 | -0.23 | 0.043 | 0.005 | 9.00 | <0.001 | 0.25 | 0.32 |
|  | V-M | 264 | -0.10 | 0.044 | 0.007 | 6.32 | <0.001 | 0.07 | 0.63 |
|  | E-N | 264 | -0.87 | 0.061 | 0.010 | 6.19 | <0.001 | 0.09 | 0.63 |
|  | E-S | 263 | -1.24 | 0.074 | 0.006 | 12.67 | <0.001 | 0.43 | 0.47 |
|  | A-H | 263 | -0.27 | 0.042 | 0.006 | 6.87 | <0.001 | 0.13 | 0.40 |
|  | A-L | 263 | -0.59 | 0.046 | 0.009 | 5.36 | <0.001 | 0.11 | 0.40 |
| **TMAX** | V-L | 264 | -3.89 | 0.417 | 0.022 | 18.80 | <0.001 | 0.57 | 0.57 |
|  | V-M | 264 | 0.77 | 0.116 | 0.023 | 5.14 | <0.001 | 0.06 | 0.49 |
|  | E-N | 264 | 4.40 | 0.116 | 0.032 | 3.70 | <0.001 | 0.02 | 0.80 |
|  | E-S | 263 | 0.18 | 0.287 | 0.032 | 9.02 | <0.001 | 0.15 | 0.52 |
|  | A-H | 263 | -2.78 | 0.243 | 0.029 | 8.27 | <0.001 | 0.21 | 0.21 |
|  | A-L | 263 | -3.63 | 0.195 | 0.039 | 4.97 | <0.001 | 0.10 | 0.29 |
| **Cooling** | V-L | 264 | -0.17 | 0.030 | 0.003 | 10.52 | <0.001 | 0.33 | 0.33 |
|  | V-M | 264 | 0.12 | 0.005 | 0.002 | 2.44 | 0.0154 | 0.02 | 0.08 |
|  | E-N | 264 | -0.05 | 0.009 | 0.002 | 4.08 | <0.001 | 0.05 | 0.51 |
|  | E-S | 263 | -0.43 | 0.025 | 0.003 | 8.75 | <0.001 | 0.17 | 0.57 |
|  | A-H | 263 | -0.19 | 0.012 | 0.003 | 4.82 | <0.001 | 0.08 | 0.28 |
|  | A-L | 263 | -0.22 | 0.009 | 0.003 | 2.94 | 0.0036 | 0.04 | 0.14 |
